# The efficiency of bio-char as bitumen modifier

**DOI:** 10.1016/j.heliyon.2023.e23192

**Published:** 2023-12-06

**Authors:** Valeria Loise, Pietro Calandra, Alfonso Policicchio, Luigi Madeo, Cesare Oliviero Rossi, Michele Porto, Abraham Abe, Raffaele G. Agostino, Paolino Caputo

**Affiliations:** a University of Calabria, Department of Chemistry and Chemical Technologies, Via P. Bucci Cubo 14D, 87036, Rende, CS, Italy; b National Research Council, CNR-ISMN, Via Salaria km. 29.300, 00015, Monterotondo, Stazione, RM, Italy; c University of Calabria, Department of Physics, Via Ponte P. Bucci, Cubo 31C, 87036, Arcavacata di Rende, CS, Italy; d CNISM - National Interuniversity Consortium for the Physical Sciences of Matter, Via della Vasca Navale, 84, 00146, Rome, Italy; e CNR-Nanotec, c/o Università della Calabria, Via P. Bucci, Cubo 31C, 87036, Arcavacata di Rende, CS, Italy

**Keywords:** Biocompatible additive, Chemical structure, Bitumen, Char, Activated carbon, Rheology, NMR

## Abstract

Improving the mechanical properties of bitumen is an important goal for road pavements design. For this reason, new compounds are now being sought for testing as bitumen modifiers.

In this work, the authors studied the effect that two different chars have on two 50/70 bitumens with different chemical and physical characteristics. A complete morphological, surface and bulk characterization of the two additives was carried out. In addition, rheology, Nuclear Magnetic Resonance (NMR) relaxometry and atomic force microscopy were used to analyze the effect that the two additives exert on the properties of the bitumens. According to the results, the char sample with high porosity could be used as a modifier of mechanical properties, while no rejuvenation effects were observed for either of the two additives tested. In addition, the two additives do not give rise to segregation phenomena.

## 1 Introduction

In recent years, the increased interest towards natural resources, that can emulate well known properties of synthetic chemical molecules, strongly influenced the scientific research. This goal has driven the research in the direction of reusing of raw materials and an enhancement of waste products [[1], [2], [3], [4], [5], [6]]. In this perspective, the design of highly performing road pavements allows an extension of the useful life of the asphalt with a consequent impact in environmental and economic terms [[7], [8], [9]]. In fact, the design of durable and more performing roads allows both a reduction in the production of Reclaimed Asphalt Pavement - RAP, i.e. the material coming from the milling of the road surface course, and a reduction in the use of raw materials.

Moreover, it would be advisable to design road pavements using environmentally friendly materials. In this regard, biomass is a widely available renewable resource [10]. Specifically, bio-char obtained as pyrolysis solid by-product of biomass turns out to be a very promising material [11,12]. Pyrolysis is a thermo-chemical degradation process that occurs in absence of oxygen from which it is possible to obtain two other fractions, namely oil and gas [[13], [14], [15], [16]]. Bio-char can be defined as “Black Carbon”, a material rich in carbon; however, it does not belong to the excluded forms of Black Carbon deriving from non-renewable sources, i.e. fuels fossils [[17], [18], [19]]. It is an *activated carbon*, obtained by heating at high temperatures for long times (hours) a based carbon materials, usually in presence of reagents and used in industrial processes for filtration, cleaning, adsorption of gases, liquids and contaminants [20].

As reported by Lu et al., nanostructured carbons are extremely promising for application as infrastructure materials [21]. Referring to bituminous materials for road paving application, bio-char particles are optimal candidates for improving their overall performances thanks to: 1) the carbonaceous nature highly compatible with the organic fraction of asphalt (bitumen) and 2) the porous/fibrous structure that allows for strong interaction with the bituminous matrix [22].

Like other powders containing micro- and nano-sized particles, which have been shown to have a beneficial effects on bitumens even at low percentages [23], bio-char allows for increased pavement loading capacity and decreased fatigue cracking during the pavement's operational life. pavement, as widely reported in the literature [[24], [25], [26]]. Anti-aging effects have also been explored. In 2021 Rajib et al. [27] tested biochar to avoid the oxidation and UV aging in bitumens, and Kumar et al. studied the thermal storage stability of binders mixed with pyrolyzed plastic waste [28]. Contrariwise, the current literature regarding the rejuvenating properties of bio-char is very limited, an indication of the scarce experimental activity in this regard. Recently, a summarized description of studies regarding the use of biochar in asphalt binders and asphalt mixes is reported in a short critical review paper by Rondón-Quintana et al. [29]. Indeed, the studies main parts deals with the use of biochar as a cement substitute in mortar or concrete. In bitumen, bio-char has shown an increase in viscosity and stiffness, improving their resistance to permanent deformation (rutting). Moreover, as reported by Ma et al. [30], the modification mechanism and structural changes of the bituminous colloidal system following modifications with biochar remain to be understood.

Our research work aims to exploit the potential of bio-char as an additive for bitumen, investigating both the modifying and rejuvenating effects, trying to analyze more deeply the action that bio-char exerts on the inner structures of the bitumen.

In order to have a more complete picture of the situation, in this experimental work two different types of commercial bio-char have been analysed as modifiers and rejuvenators of bituminous materials. The experimental work is divided as follows:

1) two different 50/70 penetration grade bitumens were considered. To each of them, two bio-chars (whose particles size, pore size distribution and mesostructure were determined by porosimetry and SEM) were added.
2) The resulting modified bituminous samples were then characterized by rheometry, NMR, and AFM [31] and compared to pristine bitumens, providing information both on the structure and on the overall performances, giving a framework of utmost importance for future efficient production of bio-char and application [32].

## 2 Materials and methods

Two 50/70 penetration grade bitumens with different Penetration Grade, Softening Points and Asphaltene contents were used for the present research (see Table 1 for their characteristics), one labelled as MOL and supplied by Polyglass s.p.a. (Italy), the other one labelled as LP and supplied by Lo Prete Costruzioni (Italy). Both bitumens were modified with two commercial bio-char, Nuchar SA-1500 (MeadWestvaco Speciality Chemicals - USA) and Filtercarb PHA (Carbon Italia - Italy) (see Table 2), hereinafter labelled as Nuchar and PHA. Samples were prepared by adding 6 % by weight of bio-char to the hot bitumen (∼150 °C) and mixed by mechanical stirrer (RW 20 Digital, IKA, Germany), around 700 rpm, for 30 min according to the protocol reported in literature [33]. The use of a temperature probe assured temperature stability during mixing. Moreover, in order to evaluate the potentialities of these bio-char as rejuvenator, both bitumens were: i) aged through Rolling Thin Film Oven Test according to ASTM D2872-04 extended to 225 min [34] instead of the normally adopted 75 min. This new protocol ensures to obtain a bitumen rigid enough to simulate a prolonged ageing process of about 10–12 years, which is a period typical of recycled asphalts, as already done in previous papers [35]; ii) modify with 6 % of bio-char. Preliminary investigations allowed us to choose this percentage as optimal dosage for our goal. Furthermore, since in general the nanoparticles are subject to segregation phenomena within the bituminous matrix [36,37], the thermal storage stability of modified bitumens was investigated, according to ASTM D5892. The modified bitumens was poured into aluminium tubes (l = 140 mm, ø = 25 mm) and placed vertically in an oven at the temperature of 163 °C for 48 h [38]. Subsequently, the samples were kept in a freezer at a temperature of −7 °C for 4 h, and the upper and lower portion were analysed [39].

**Table 1:** Information about the investigated bitumens.

Label	Penetration Grade (0.1 mm) ± 1	Softening Point (°C) ± 0.2	Asphaltene content (%) ± 0.5
MOL	68	48.8	26.8
LP	63	55	30.9

**Table 2:** Information about the biochar.

Label	Density g/cm^3^	Declared SSA m^2^/g
Nuchar SA-1500	0.33	2139
Filtercarb PHA	0.37	1100–1200

Analyses on the two biochar-treated bitumen samples were performed in triplicate to ensure the reproducibility of the results.

### 2.1 Bio-char characterization

#### 2.1.1 Surface and bulk characterization

Porosity was evaluated through Nitrogen adsorption isotherm on ASAP 2460 (Micromeritics) at 77 K. Before the experiments, the samples were outgassed at 200 °C until a constant vacuum of 10^−7^ mbar was reached. BET method was used to calculate the specific surface area (S_BET_), while the volume of micropores (V_mic_), volume of mesopores (V_mes_), total pore volume (V_T_), and pore size distribution (PSD) were calculated by Non-local Density Functional Theory (NLDFT) [40], with the assumption of the pore wall heterogeneity.

#### 2.1.2 Morphological characterization

Scanning electron images were acquired using a Quanta FEG 400 (FEI) scanning electron microscope (SEM). All images, that provide typical macro e meso-scale morphology of the analysed samples, were recorded by using an electron beam of 15 keV.

### 2.2 Bitumen characterization

#### 2.2.1 Rheology

Rheological measurements were carried out to evaluate the effects of the two biochars exert on the visco-elastic properties of the bitumens. A certain amount of sample was placed on the Rheomether's Peltier plate and heated to 100 °C. Subsequently, the sample was allowed to cool to 20 °C and conditioned for 600 s. This operating procedure ensures that the thermal history of the material is erased [34,41].

Rheological measurements were carried out using a shear stress-controlled rheometer SR5 (Rheometric scientific, USA) equipped with a parallel plate geometry (gap 2 mm, diameter 25 mm) in the temperature range 20–140 °C and a Peltier system (±0.1 °C) for temperature control. Dynamic experiments were performed within the linear viscoelastic region where measured material features are independent of the amplitude of applied load and are the only function of microstructure of material [42]. This analysis makes it possible to monitor the trend of the elastic and viscous modulus as the temperature varies. Since the modules are related to each other through the equation.

tanδ=G″G′ , where $G^{\prime \prime }$ is the viscous modulus and $G^{\prime }$ is the elastic modulus, when the bitumen loses its elastic component it becomes a viscous fluid and the tangent diverges. The temperature corresponding to the loss of the elastic component is defined as the sol transition temperature.

#### 2.2.2 NMR relaxometry

In order to evaluate the effect of the bio-char on the inner structure of the analysed bitumens, NMR relaxometry experiments were carried out. Based on the spin-spin relaxation time, it is possible to evaluate differences on the maltene components of the samples. In fact, the signals provide information about the protonic self-assembling structures. They also help to distinguish between different types of aggregations in the bitumen structure, because the protons in different types of supramolecular organization realign at different rates and produce distinct signals. Analysis were conducted with a custom-built NMR instrument, that operates at a proton frequency of 15 MHz. Analyses were performed at 15 °C below the transition temperature of each sample, for comparative purposes among the various samples [43]. This analysis was performed on the bitumen samples as it, i.e. without being dissolved in any solvent.

#### 2.2.3 Atomic force microscopy

AFM was carried out to investigate the morphology of the samples. This is a non-destructive technique that does not require any kind of sample pretreatment. A bitumen sample was heated in an oven at 100 °C for 10 min and then left to cool down at room temperature. AFM characterization was performed in tapping mode at room temperature in air on a Multimode 8. The AFM system equipped with a Nanoscope V controller (Bruker) provided simultaneous topography and phase imaging of the sample [44]. All the measurements were performed using probes with a conical tip of nominal end radius 10 nm and a resonance frequency of 150 kHz.

## 3 Results and discussion

### 3.1 Porosity

**Fig. 1:**
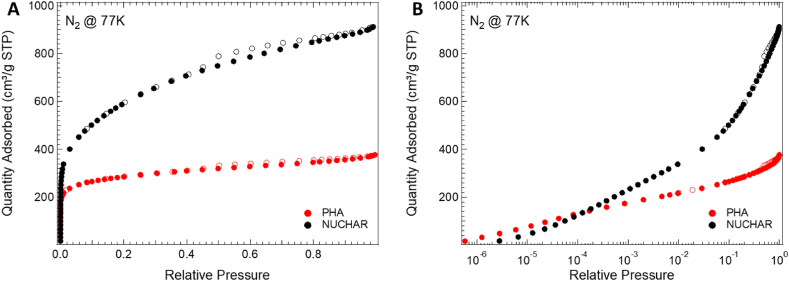
Nitrogen adsorption isotherms in A) linear and B) logarithmic scale.

Fig. 1 A) and B) shows the nitrogen adsorption/desorption isotherms acquired on the Nuchar and PHA samples at liquid nitrogen temperature. According to the IUPAC classification [45], the shapes of the isotherms are Types I and Type IV respectively. For both samples, the coexistence of both meso- and micropores is clearly visible. Looking deeper in details to the acquired isotherms, it is noteworthy that for both samples the N_2_ uptake rapidly increased at a very low relative pressure (P/P_0_ < 0.01), an indication of microporous character; in particular the Nuchar sample shows a greater amount of adsorbed N_2_ at low pressures, which is characteristic of a higher microporosity compared to PHA.

Moving to higher pressure, an indication of their micropore capacity is connected to changes in the trend and/or slope of both isotherms for pressure P/P_0_ > 0.1. The hysteresis loop in the pressure range of 0.5–1.0 is more evident for the PHA sample (see difference between open and closed points in Fig. 1 A) and B)) and is associated to the capillary condensation that occurred in mesopores. In particular, the Nuchar sample shown a narrower isothermal knee than the PHA sample, indicating mainly microporous structures with a lower mesoporosity.

**Fig. 2:**
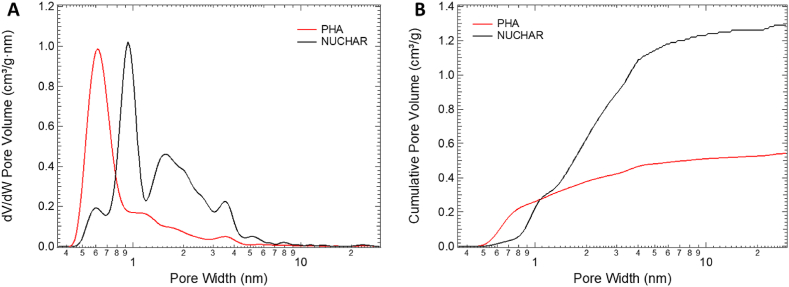
A) Pore size distributions and B) cumulative pore volumes of the sample tested.

Table 3 summarizes structural differences calculated by analysing adsorption isotherms while Fig. 2 A) and B) shows the pore size distribution (PSD) and cumulative pore volume evaluated using NLDFT. The higher cumulative pore volume for the Nuchar sample is evident.

**Table 3:** Samples textural properties calculated from the adsorption isotherms.

Sample	S_BET_ [m^2^/g]	V < 0.7 nm [cm^3^/g]	0.7 nm < V < 2 nm [cm^3^/g]	V_mic_ [cm^3^/g]	V_mes_ [cm^3^/g]	V_T_ [cm^3^/g]	V_mic_/V_T_ [%]
Nuchar SA-1500	2047	0.032	0.606	0.637	0.657	1.294	49
Filtercarb PHA	1036	0.169	0.209	0.379	0.162	0.541	70

All the PSD plots (see Fig. 2A) shown a significant peak in the ultra-microporous region (<0.7 nm) centred at 0.6 nm for the PHA sample while it is shifted to the super-micropore region (0.7 < x < 2 nm) and centred at 1 nm for the Nuchar sample. For both samples, although higher in the case of Nuchar, a series of peaks are present in the microporous region.

### 3.2 Scanning Electron Microscopy (SEM)

**Fig. 3:**
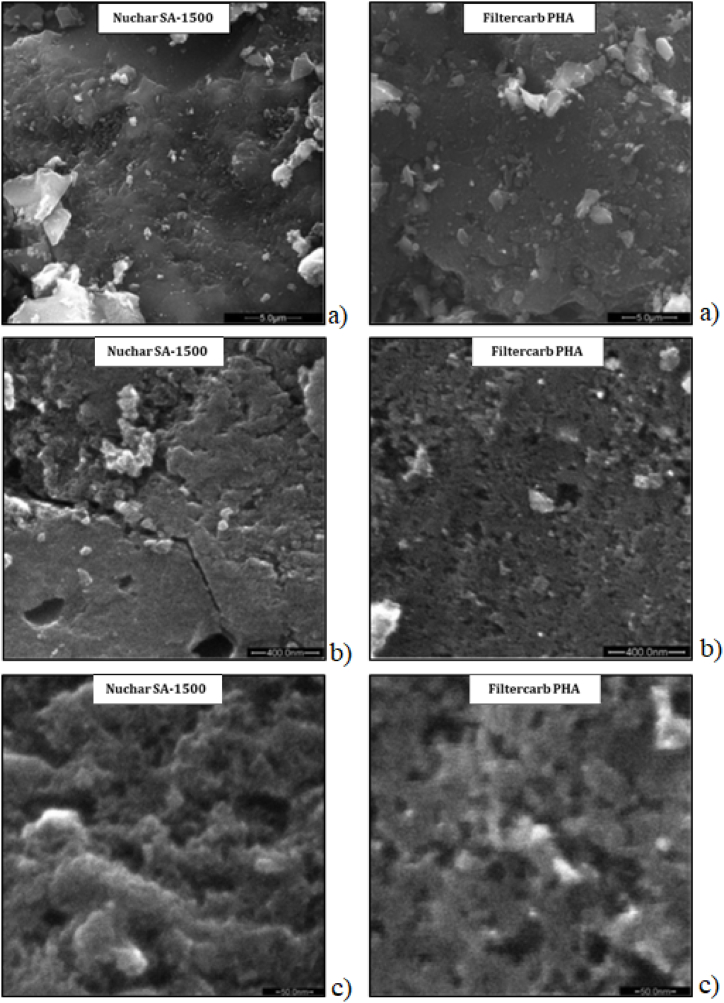
SEM images of Nuchar SA-1500 (left) and Filtercarb PHA (right) on a 5.0 μm scale (a); 400 nm scale (b) and 50 nm scale (c).

SEM images of Nuchar and PHA samples, both supplied as powders, are shown in Fig. 3 with different magnification: 8kx top panels, 120 kx center panels and 600 kx bottom panels. Looking at a lower magnification (Fig. 3a), both samples show a rather uniform surface with slight roughness over the entire area. Descending to higher magnification, the PHA sample shows uniform porosity with pore widths on the order of several tens of nanometers (see Fig. 3c). On the contrary, at higher magnification, the Nuchar sample surface appears different showing irregular and not well-defined mesopore structure.

### 3.3 Mechanical behaviour

**Fig. 4:**
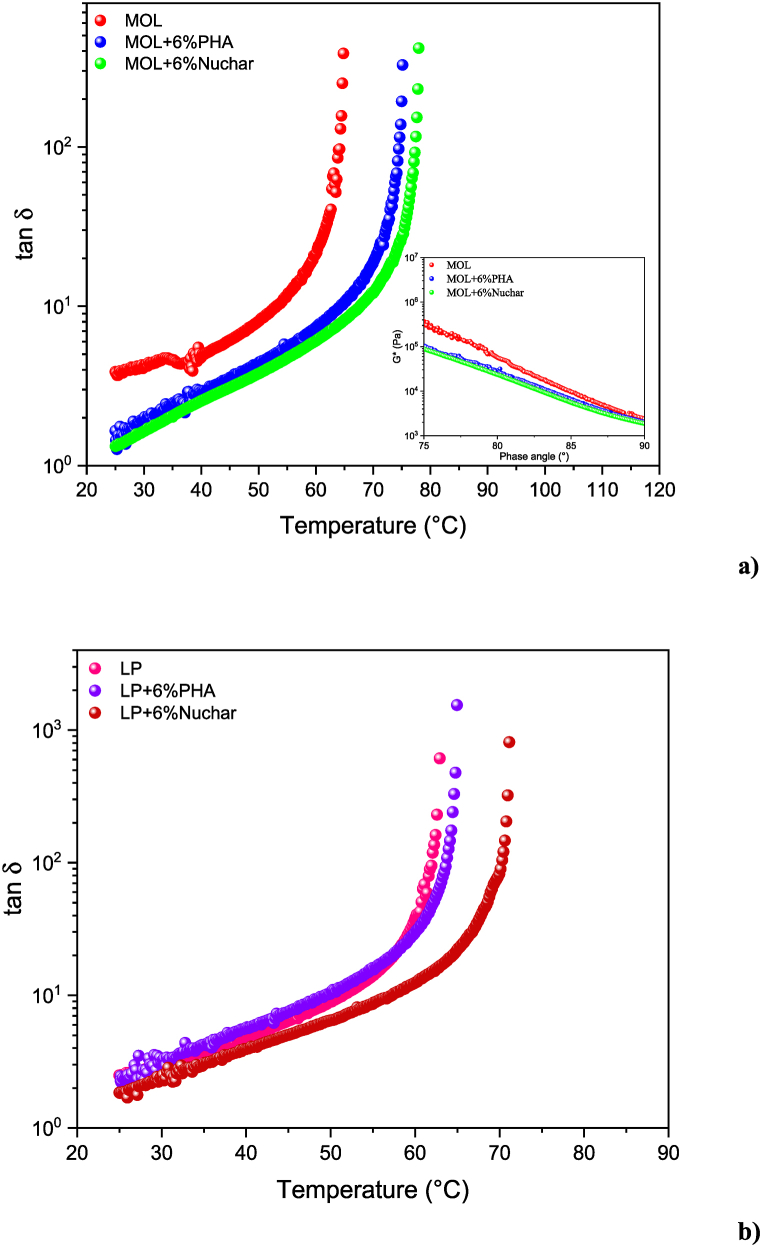
Temperature sweep (time cure) tests performed on the MOL a) and LP b) modified and unmodified.

Through temperature-sweep measurements the loss tangent (tan δ = G’‘/G’) behaviour is recorded during a temperature ramp at a constant heating rate of 1 °C per minute and at a frequency of 1 Hz, in order to observe the shift in transition temperature from viscoelastic to liquid. Fig. 4 a) and b) shows the time cure tests of both the pristine and modified bitumens with the two types of bio-char.

**Fig. 5:**
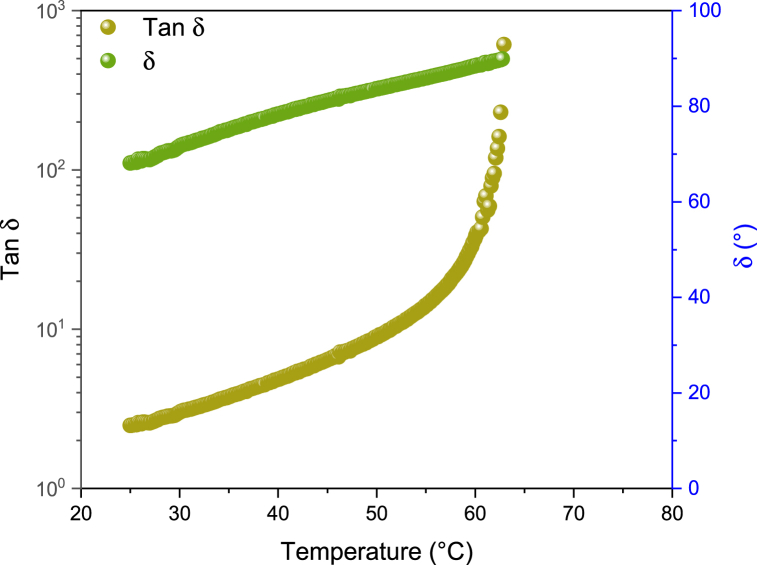
Comparison between phase angle δ and tan δ.

Increasing temperature, the analysed samples become progressively softer with G′ decreasing more quickly than G″ therefore causing a parallel increase of tan δ. For sufficiently high temperatures, at some point G′ suddenly drops so that sample can no longer store energy. This is revealed by the steep increase in tan δ. For higher temperatures, the binder behaves like a Newtonian fluid [46]. Microscopically, for temperatures above this transition temperature (T*) the molecular thermal agitation, and consequently the molecular relaxation rate, is high enough to allow the system to adapt to mechanical distortion/perturbation. This gives purely flowing behaviour and causes any elastic storage of mechanical energy to vanish (tan δ → ∞). The transition Temperature T* is determined when the phase angle (δ) arrives at 90° (see Fig. 5) or, when the G’‘/G’ > 10^3^.

The effect of the two different additives is roughly the same in the case of MOL bitumen (see Fig. 4a): both bio-char provided a hardening effect with a shift of T* of about 10 °C. However, the major shift is caused by the addition of Nuchar (about 13 °C). Instead, the hardening effect is negligible in the case of LP (see Fig. 4b) modified with PHA, conversely Nuchar allows a shift in the transition temperature about 6 °C.

Moreover, the mechanical performance of the samples was also analysed in term of Black diagram. A plot of complex modulus G* versus the phase angle δ which allows to eliminate the dependence on temperature and frequency [35].

The Black diagram, shown as insert in Fig. 4 a), highlights how the same complex modulus value is associated with decreasing phase angle values in the modified bitumen samples. This means that, for a given G*, the elastic character of bio-char kicks in. In addition, the trend of bitumen modified with the two different bio-chars is extremely similar, consistent with what was obtained by temperature sweep measurements.

From rheological measurements the rutting parameter, defined as G*/sin δ, can be calculated. The values meet the limits imposed by the Superior Performing Asphalt Pavements method under the Strategic Highway Research Program (Superpave SHRP) [47] being always higher than 1 kPa for unaged samples. The rutting parameter at 50 °C represents the mechanical property specifically under usage conditions [48]. The added filler changes this value according to its reinforcing effect on the bitumen microstructure. Fig. 5 shows all values and their correlation with T*.

**Fig. 6:**
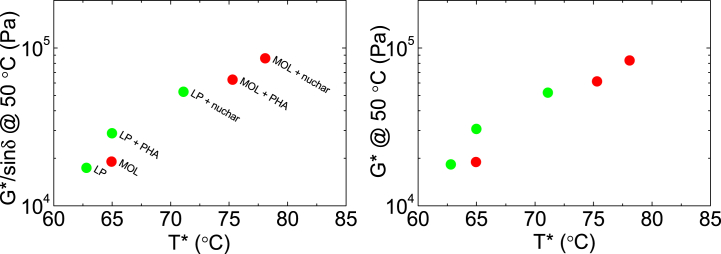
Correlation between the rutting parameter at 50 °C and the transition temperature T*.

Looking at Fig. 6 in more detail, it is noteworthy to note the parallel increase in both temperature T* and the rutting parameter at 50 °C (mechanical property), highlighting the strengthening effect caused by bio-char. Even if apparently independent, both can be seen as different aspects of the same microscopic phenomenon in which the bio-char helps to hold up the overall structures facing the disordering effect of temperature. The same correlation is shown by G* (data shown in the inset of Fig. 4a), reinforcing the above observations.

**Fig. 7:**
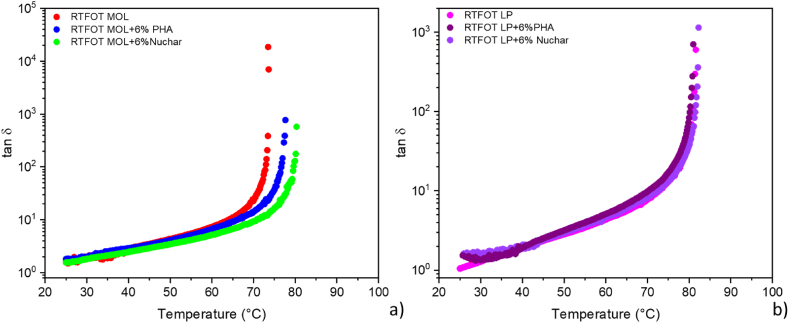
Temperature sweep (time cure) tests performed on a) aged modified and b) unmodified bitumens.

Fig. 7 shows the effect of the two bio-char on the aged bitumens. It is noticeable that both PHA and Nuchar have no effect on LP aged bitumen (see Fig. 7b), and even on MOL bitumen, they are unable to restore the viscoelastic properties of aged bitumen. Consequently, bio-chars have no rejuvenating effect but rather exert the same stiffening effect observed for fresh bitumen (Fig. 7a), to a lesser extent for LP bitumen than for MOL bitumen. The more pronounced effect exerted by Nuchar may reasonably due to its higher cumulative pore volume, compared with PHA, which allows for more extensive char-bitumen interfacial interactions involving, as a result, a larger fraction of the bitumen molecules.

**Fig. 8:**
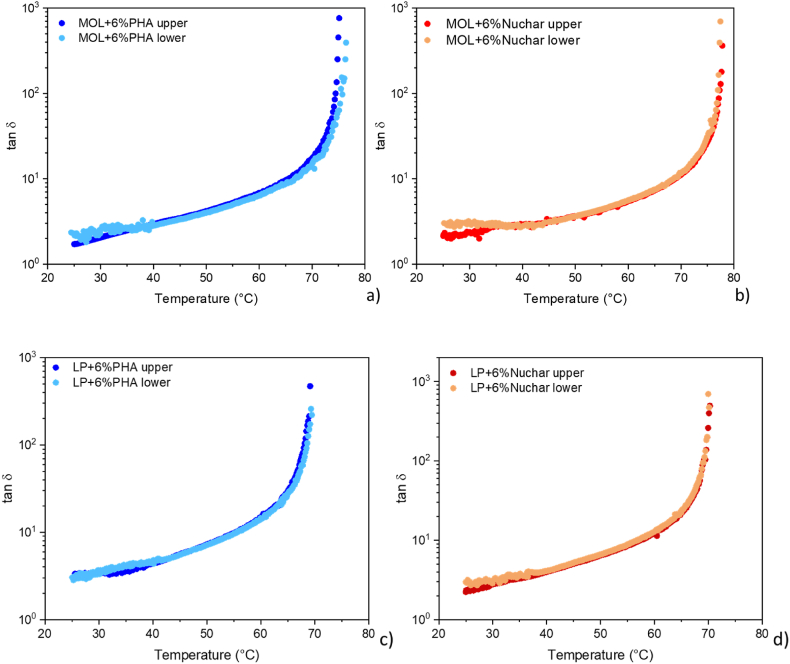
Evaluation of storage stability for MOL (a,b) and LP bitumen (c,d).

Finally, according to the results of tube test shown in Fig. 8(a–d), a good dispersion of the two products within the bituminous matrix can be observed, in fact the time cure carried out on the upper portion is comparable to that of the lower portion.

### 3.4 NMR results

**Fig. 9:**
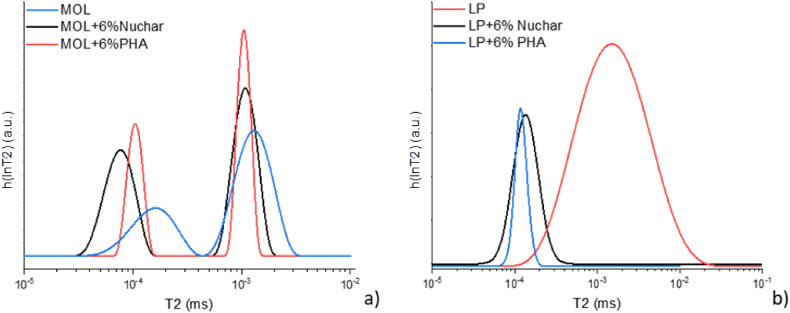
a) T_2_ distribution of MOL modified with Nuchar and with PHA and b) T_2_ distribution of LP modified with Nuchar and with PHA.

Relaxometry experiments were performed to evaluate both the modifying action and rejuvenating effect of the two additives on bitumen under examination. Fig. 9a shows two peaks for each sample analysed, in line with previous studies [49].

The first peak, at smaller T_2_ values, can be referred to asphaltenes, while the second one, with larger T_2_ values, is related to maltene phase [50]. NMR analyses performed on MOL bitumen confirm the hardening effect of the bio-char used, in fact, the relaxation times of modified bitumen drop to lower values than those of pristine bitumen. Furthermore, it appears that Nuchar has a slightly more hardening effect than PHA, due to the asphaltenic component. In fact, the maltenic portions have substantially the same relaxation times, while the asphaltenic portion of the Nuchar-modified bitumen has lower relaxation times than that of the PHA-modified bitumen. The slightly stronger effect induced by Nuchar compared with PHA agrees with the rheological clues (see T* and rutting parameter data).

LP bitumens, on the other hand, show a unique distribution of relaxation times as shown in Fig. 9b. This is because the relaxation times of each components, i.e. asphaltenes and maltene, are extremely close and the Laplace Inverse cannot resolve them distinctly. However, a treatment in terms of average values is possible, noting that the two additives have approximately the same effect on pristine bitumen, causing it to harden slightly.

**Fig. 10:**
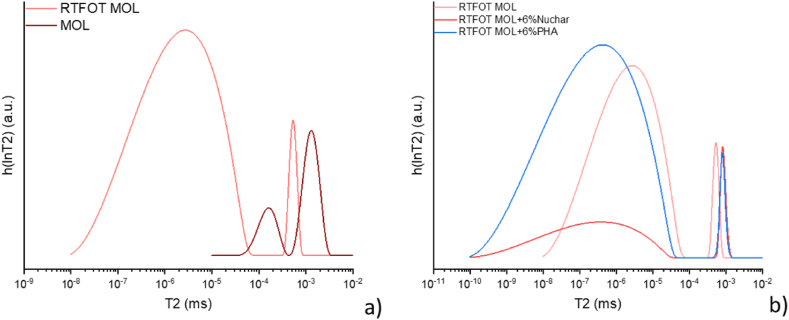
a) Comparison between T2 distribution of MOL and RTFOT MOL bitumen and b) RTFOT MOL modified bitumen.

NMR analyses carried out on aged bitumen confirm that the two additives do not have effect as rejuvenating agents. In fact, a rejuvenating agent should restore the inner micro-structures of the bitumen by restoring the right asphaltene/maltene balance [51]. In this case, the two additives tested do not perform this function. Looking at Fig. 10a it can be deduced that the T_2_ referred to the asphaltenic fraction are of the order of 10^−4^, while those of the maltene component are of the order of 10^−3^. On the other hand, in modified aged samples the relaxation times of the asphaltenic component are of the order of 10^−6^, while those of the maltene component are of the order of 10^−4^/10^−3^ (Fig. 10b). Indeed, PHA and, to a lesser extent, Nuchar continue to have a hardening effect on RTFOT aged MOL bitumen, as demonstrated by rheological experiments.

**Fig. 11:**
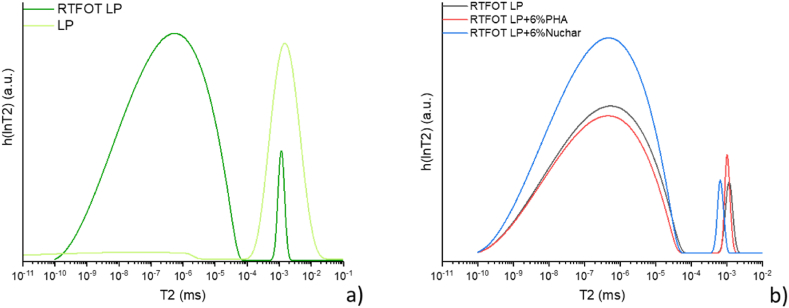
Comparison between a) T_2_ distribution of LP and RTFOT LP bitumen and b) RTFOT LP modified.

With reference to LP bitumen aged by RTFOT, two distributions of relaxation times are observed (see Fig. 11a). The increase in the asphaltenic fraction in the aged bitumen caused the difference in the relaxation times of the maltenic and asphaltenic fractions to increase to such an extent that it could be solved by the Laplace inverse transform [52] and thus obtain two distinct peaks. Again, the two additives have limited effect on aged bitumen (see Fig. 11b).

### 3.5 Atomic force microscopy (AFM)

**Fig. 12:**
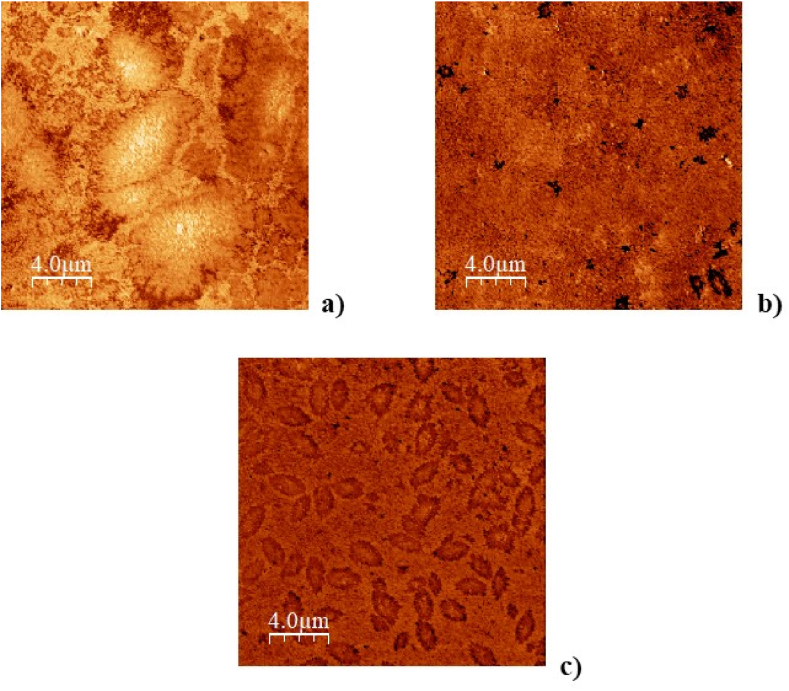
a) AFM phase images of MOL unmodified, b) modified with PHA and c) Nuchar.

**Fig. 13:**
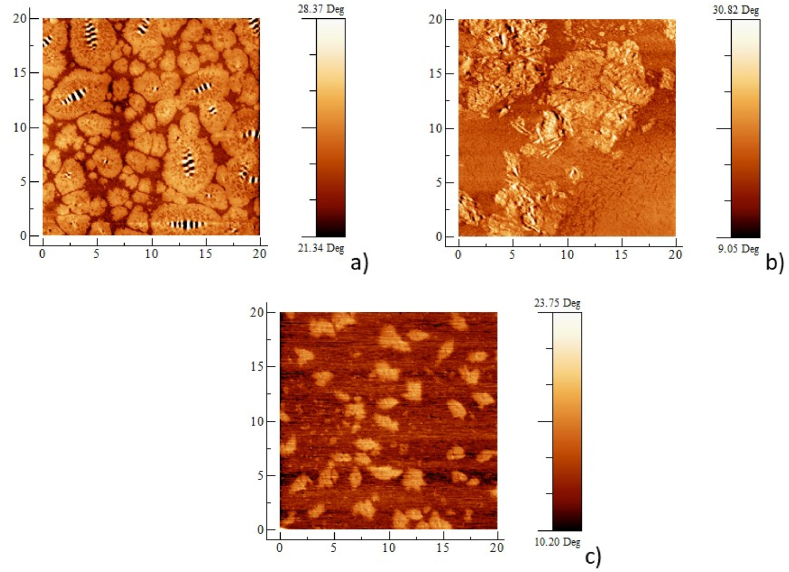
AFM phase images of LP unmodified a) and modified with PHA b) and Nuchar c).

Because the two additives were found to be modifiers and not rejuvenators, AFM analyses were performed only on unaged bitumen samples. Each acquisition was performed at room temperature. The AFM measurements on MOL and LP bitumen are shown in Fig. 12, Fig. 13. The images (see Fig. 12, Fig. 13a) reveal that both bitumens are characterized by the presence of large domains strongly interconnected. The average size of the domains in the case of MOL is about 7 μm. In contrast, for LP bitumens in general, the domains are slightly smaller (about 6.6 μm), because they are mediated by the presence of both large and smaller domains (see Fig. 13 a). It should be noted that the micro-scale domains observed are made up of self-assembled asphaltene molecules in hierarchical structures at various lengthscales hold [53] up by interactions of various strengths occurring at different levels of complexity [54]. The so-called *bee* structure [55], which is clearly visible in Fig. 13a, is one of the detectable consequence of this self-assembly [56]. In this framework, the effect of Nuchar is evident (see Fig. 12, Fig. 13c), in fact it manages to break up the asphaltenic structures self-assembled at the micro-scale, having an effect similar to that of PPA [57]. Indeed, the size of the domains is reduced to about 2.2 μm in the case of MOL bitumen and to about 1.6 μm in the case of LP bitumen. As reported in other studies [58], the formation of smaller and evenly distributed asphaltenes within the matrix manages to stabilize the system by improving its properties. The fact that such aggregates turns out to be evenly distributed can be seen as the effect of the amphiphilic components (resins) present in the bitumen. In fact, amphiphiles have proven to be able to stabilize both organic [59] and inorganic [60] clusters in apolar media, an effect in accordance with the micellar model of bitumens [61,62]. As for PHA, on LP bitumen the aggregation effect it is evident (see Fig. 13b) where large clusters (about 9 μm) form a colloidal network, according to literature data [58] the melt transition is similar to that one of the pristine bitumen as expected. In Fig. 13b there appears to be the presence of small asphaltenes although the low contrast of the image makes it difficult to distinguish the aggregates very well.

## 4 Conclusions

The modifying and rejuvenating effects of two commercial chars (Nuchar SA-1500 and Filtercarb PHA) were explored on two bitumen samples. As evidenced by rheometry, both chars strengthen the two bitumens, with a more pronounced effect exerted by Nuchar, reasonably due to its higher cumulative pore volume. However, none of them shows significant rejuvenating effects.

These clues were confirmed both from the dynamical (by NMR-relaxometry measurements - T_2_ distibutions), and by the structural (by Atomic Force Microscopy) point of view, providing a self-consistent multi-aspect picture of the microscopic, molecule-based phenomena/processes involved, where the nature of carbon char and porous structure are able to reinforce the overall intermolecular network in bitumens but are unable to confer rejuvenation effects by destroying the strong self-assembled asphaltenic aggregates typical of aged bitumens. The work provides important data highlighting the microscopic effects induced by two common chars for future pilot design of ad-hoc additives in bitumens. This study is a preliminary investigation of the char/bitumen.

It is now known from both theoretical and experimental studies that nanoparticles have significant effects on the performance of asphalt binders [63]. The raw materials for biochar production are often biomass wastes generated from crop harvesting, agroforesty, animal waste, municipal waste, etc [64]. Biochar can have different origins making this material heterogeneous. This means that biochar from different sources shows different elemental composition, functional groups, structural properties and so on [65]. Nevertheless, the use of char is crucial in terms of the circular economy, and the possibility of its use in the bitumen or pavement sector in general may open up a new scenario. Of course, subsequent more in-depth investigations involving different chars and bitumens of different penetration grades are needed to understand the correlation between mechanical properties and the char morphologies. In addition, it would be desirable to understand what specific characteristics the biochar must have to improve bitumen performance, i.e., specific functional groups, specific dimensions, porosity, surface area/volume ratio, etc.

## Funding

This research was funded by @CNR Project ReScA, “Recupero degli scarti da pirolisi di rifiuti urbani per potenziare e ripristinare asfalti,” decision of Administration Council dated 21 Dicembre 2021.

## Data availability

Has data associated with your study been deposited into a publicly available repository? No.

Has data associated with your study been deposited into a publicly available repository?

Data will be made available on request.

## CRediT authorship contribution statement

**Valeria Loise:** Data curation. **Pietro Calandra:** Formal analysis. **Alfonso Policicchio:** Resources. **Luigi Madeo:** Investigation. **Cesare Oliviero Rossi:** Conceptualization. **Michele Porto:** Investigation. **Abraham Abe:** Investigation. **Raffaele G. Agostino:** Data curation. **Paolino Caputo:** Investigation, Data curation.

## Declaration of competing interest

The authors declare that they have no known competing financial interests or personal relationships that could have appeared to influence the work reported in this paper.

## References

[1] Lehmann J., Joseph S. (2015). Biochar for Environmental Management: Science, Technology and Implementation.

[2] Porto M., Caputo P., Loise V., Abe A.A., Tarsi G., Sangiorgi C., Gallo F., Oliviero Rossi C. (2021). Preliminary study on new alternative binders through Re-refined engine oil bottoms (REOBs) and industrial by-product additives. Mol.

[3] Aprianti E. (2016). A huge number of artificial waste material can be supplementary cementitious material (SCM) for concrete production – a review Part II. J. Clean. Prod..

[4] Zhou K., Gong K., Zhou Q., Zhao S., Guo H., Qian X. (2020). Estimating the feasibility of using industrial solid wastes as raw material for polyurethane composites with low fire hazards. J. Clean. Prod..

[5] Zakrevskaya L., Gavrilenko A., Andreeva K., Lubin P., Kapush I., Udin I. (2020). Man-made soils and mining wastes as raw materials for building composites. IOP Conf. Ser. Mater. Sci. Eng..

[6] Gunture J. Kaushik, Garg A.K., Saini D., Khare P., Sonkar S.K. (2020). Pollutant diesel soot derived onion-like nanocarbons for the adsorption of organic dyes and environmental assessment of treated wastewater. Ind. Eng. Chem. Res..

[7] Sollazzo G., Longo S., Cellura M., Celauro C. (2020). Impact analysis using life cycle assessment of asphalt production from primary data. Sustain. Times.

[8] Caputo P., Abe A.A., Loise V., Porto M., Calandra P., Angelico R., Oliviero Rossi C. (2020). The role of additives in warm mix asphalt technology: an insight into their mechanisms of improving an emerging technology. Nanomaterials.

[9] Cao R., Leng Z., Hsu S.-C. (2019). Comparative eco-efficiency analysis on asphalt pavement rehabilitation alternatives: hot in-place recycling and milling-and-filling. J. Clean. Prod..

[10] Caputo P., Calandra P., Loise V., Le Pera A., Putz A.-M., Abe A.A., Madeo L., Teltayev B., Luprano M.L., Alfè M., Gargiulo V., Ruoppolo G., Rossi C.O. (2022). When physical chemistry meets circular economy to solve environmental issues: how the ReScA Project aims at using waste pyrolysis products to improve and rejuvenate bitumens. Sustain. Times.

[11] Degirmen G., Hande B. (2022).

[12] Engineering M. (2013). Applied Surface Science Adsorption of heavy metal ions from aqueous solutions by bio-char , a by-product of pyrolysis. Appl. Surf. Sci..

[13] Guizani C., Jeguirim M., Valin S., Limousy L., Salvador S. (2017). Biomass chars: the effects of pyrolysis conditions on their morphology, structure, chemical properties and reactivity. Energies.

[14] Panwar N.L., Kothari R., V Tyagi V. (2012). Thermo chemical conversion of biomass – eco friendly energy routes. Renew. Sustain. Energy Rev..

[15] Sharma A., Pareek V., Zhang D. (2015). Biomass pyrolysis—a review of modelling, process parameters and catalytic studies. Renew. Sustain. Energy Rev..

[16] Yang R., Kang S., Ozer H., Al-Qadi I.L. (2015). Environmental and economic analyses of recycled asphalt concrete mixtures based on material production and potential performance. Resour. Conserv. Recycl..

[17] Lehmann J., Gaunt J., Rondon M. (2006). Bio-char sequestration in terrestrial ecosystems – a review. Mitig. Adapt. Strategies Glob. Change.

[18] Keiluweit M., Nico P.S., Johnson M.G., Kleber M. (2010). Dynamic molecular structure of plant biomass-derived black carbon (biochar). Environ. Sci. Technol..

[19] Xiao F., Bedane A.H., Zhao J.X., Mann M.D., Pignatello J.J. (2018). Thermal air oxidation changes surface and adsorptive properties of black carbon (char/biochar). Sci. Total Environ..

[20] Reza M.S., Yun C.S., Afroze S., Radenahmad N., Bakar M.S.A., Saidur R., Taweekun J., Azad A.K. (2020). Preparation of activated carbon from biomass and its' applications in water and gas purification, a review. Arab J. Basic Appl. Sci..

[21] Lu S.-N., Xie N., Feng L.-C., Zhong J. (2015). Applications of nanostructured carbon materials in constructions: the state of the art. J. Nanomater..

[22] Zhao S., Huang B., Ye X.P., Shu X., Jia X. (2014). Utilizing bio-char as a bio-modifier for asphalt cement: a sustainable application of bio-fuel by-product. Fuel.

[23] Taborda E.A., Franco C.A., Ruiz M.A., Alvarado V., Cortés F.B. (2017). Experimental and theoretical study of viscosity reduction in heavy crude oils by addition of nanoparticles. Energy Fuel..

[24] Das O., Sarmah A.K., Bhattacharyya D. (2015). Structure–mechanics property relationship of waste derived biochars. Sci. Total Environ..

[25] Zhang R., Dai Q., You Z., Wang H., Peng C. (2018). Rheological performance of bio-char modified asphalt with different particle sizes. Appl. Sci..

[26] Gan X., Zhang W. (2021). Application of biochar from crop straw in asphalt modification. PLoS One.

[27] Rajib A., Saadeh S., Katawal P., Mobasher B., Fini E.H. (2021). Enhancing biomass value chain by utilizing biochar as A free radical scavenger to delay ultraviolet aging of bituminous composites used in outdoor construction. Resour. Conserv. Recycl..

[28] Kumar A., Choudhary R., Kumar A. (2021). Characterization of thermal storage stability of waste plastic pyrolytic char modified asphalt binders with sulfur. PLoS One.

[29] Rondón-Quintana H.A., Reyes-Lizcano F.A., Chaves-Pabón S.B., Bastidas-Martínez J.G., Zafra-Mejía C.A. (2022). Use of biochar in asphalts: review. Sustainability.

[30] Ma F., Dai J., Fu Z., Li C., Wen Y., Jia M., Wang Y., Shi K. (2022). Biochar for asphalt modification: a case of high-temperature properties improvement. Sci. Total Environ..

[31] Caputo P., Porto M., Calandra P., De Santo M.P., Oliviero Rossi C. (2018). Effect of epoxidized soybean oil on mechanical properties of bitumen and aged bitumen. Mol. Cryst. Liq. Cryst..

[32] Amin F.R., Huang Y., He Y., Zhang R., Liu G., Chen C. (2016). Biochar applications and modern techniques for characterization. Clean Technol. Environ. Policy.

[33] Oliviero Rossi C., Caputo P., Loise V., Miriello D., Teltayev B., Angelico R. (2017). Role of a food grade additive in the high temperature performance of modified bitumens. Colloids Surfaces A Physicochem. Eng. Asp..

[34] Caputo P., Oliviero Rossi C. (2021). Differential scanning calorimetry as a new method to evaluate the effectiveness of rejuvenating agents in bitumens. Appl. Sci..

[35] Calandra P., Quaranta S., Apolo Miranda Figueira B., Caputo P., Porto M., Oliviero Rossi C. (2022). Mining wastes to improve bitumen performances: an example of circular economy. J. Colloid Interface Sci..

[36] Calandra P., Loise V., Porto M., Oliviero Rossi C., Lombardo D., Caputo P. (2020). Exploiting nanoparticles to improve the properties of bitumens and asphalts: at what extent is it really worth it?. Appl. Sci..

[37] Zhang W., Capco D.G., Chen Y. (2014). Nanoparticle Aggregation: Principles and Modeling BT - Nanomaterial: Impacts on Cell Biology and Medicine.

[38] ASTM, “Standard Practice for Determining the Separation Tendency of Polymer from Polymer Modified Asphalt,” (n.d.).

[39] Wang H., Liu X., Erkens S., Skarpas A. (2020). Experimental characterization of storage stability of crumb rubber modified bitumen with warm-mix additives. Construct. Build. Mater..

[40] Policicchio A., Florent M., Attia M.F., Whitehead D.C., Jagiello J., Bandosz T.J. (2020). Effect of the incorporation of functionalized cellulose nanocrystals into UiO-66 on composite porosity and surface heterogeneity alterations. Adv. Mater. Interfac..

[41] Rossi C.O., Ashimova S., Calandra P., De Santo M.P., Angelico R. (2017). Mechanical resilience of modified bitumen at different cooling rates: a rheological and atomic force microscopy investigation. Appl. Sci..

[42] Yusoff N.I.M., Shaw M.T., Airey G.D. (2011). Modelling the linear viscoelastic rheological properties of bituminous binders. Construct. Build. Mater..

[43] Bortolotti V., Brown R.J.S., Fantazzini P. (2009).

[44] You A., Be M.A.Y. (2019).

[45] Sing K.S.W., Everett D.H., Haul R.A.W., Moscou L., Pierotti R.A., Rouquerol J., Siemieniewska T. (2008). Reporting physisorption data for gas/solid systems. Handb. Heterog. Catal..

[46] Loeber L., Muller G., Morel J., Sutton O. (1998). Bitumen in colloid science: a chemical, structural and rheological approach. Fuel.

[47] Kennedy T.W., Huber G., Harrigan E.T., Cominsky R.J., Hughes C.S., Von Quintus H.L., Moulthrop J.S. (1994).

[48] Somé C. (2016).

[49] Loise V., Caputo P., Porto M., Calandra P., Angelico R., Rossi C.O. (2019). A review on Bitumen Rejuvenation: mechanisms, materials, methods and perspectives. Appl. Sci..

[50] Muhammad A., Azeredo R.B. de V. (2014). 1H NMR spectroscopy and low-field relaxometry for predicting viscosity and API gravity of Brazilian crude oils – a comparative study. Fuel.

[51] Loise V., Caputo P., Porto M., Teltayev B., Angelico R., Oliviero Rossi C. (2020). Unravelling the role of a green rejuvenator agent in contrasting the aging effect on bitumen: a dynamics rheology, nuclear magnetic relaxometry and self-diffusion study. Colloids Surfaces A Physicochem. Eng. Asp..

[52] Chen S., Wang D., Yi J., Feng D. (2019). Implement the Laplace transform to convert viscoelastic functions of asphalt mixtures. Construct. Build. Mater..

[53] Tanaka R., Sato E., Hunt J.E., Winans R.E., Sato S., Takanohashi T. (2004). Characterization of asphaltene aggregates using X-ray diffraction and small-angle X-ray scattering. Energy Fuel..

[54] Calandra P., Caputo P., De Santo M.P., Todaro L., Turco Liveri V., Oliviero Rossi C. (2019). Effect of additives on the structural organization of asphaltene aggregates in bitumen. Construct. Build. Mater..

[55] Ganter D., Franzka S., V Shvartsman V., Lupascu D.C. (2022). The phenomenon of bitumen ‘bee’’ structures – bulk or surface layer – a closer look. Int. J. Pavement Eng..

[56] Jäger A., Lackner R., Eisenmenger-Sittner C., Blab R. (2004). Identification of microstructural components of bitumen by means of atomic force microscopy (AFM). Proc. Appl. Math. Mech..

[57] Dourado E., Pizzorno B., Motta L., Simão R., Leite L. (2014). Analysis of asphaltic binders modified with PPA by surface techniques. J. Microsc..

[58] Baldino N., Gabriele D., Lupi F.R., Oliviero Rossi C., Caputo P., Falvo T. (2013). Rheological effects on bitumen of polyphosphoric acid (PPA) addition. Construct. Build. Mater..

[59] Calandra P., Giordano C., Ruggirello A., Turco Liveri V. (2004). Physicochemical investigation of acrylamide solubilization in sodium bis(2-ethylhexyl)sulfosuccinate and lecithin reversed micelles. J. Colloid Interface Sci..

[60] Calandra P., Di Marco G., Ruggirello A., Liveri V.T. (2009). Physico-chemical investigation of nanostructures in liquid phases: nickel chloride ionic clusters confined in sodium bis(2-ethylhexyl) sulfosuccinate reverse micelles. J. Colloid Interface Sci..

[61] Lesueur D. (2009). The colloidal structure of bitumen: consequences on the rheology and on the mechanisms of bitumen modification. Adv. Colloid Interface Sci..

[62] Porto M., Angelico R., Caputo P., Abe A.A., Teltayev B., Rossi C.O. (2022).

[63] Caputo P., Porto M., Angelico R., Loise V., Calandra P., Oliviero Rossi C. (2020). Bitumen and asphalt concrete modified by nanometer-sized particles: basic concepts, the state of the art and future perspectives of the nanoscale approach. Adv. Colloid Interface Sci..

[64] Wani I., Ramola S., Garg A., Kushvaha V. (2021). Critical review of biochar applications in geoengineering infrastructure: moving beyond agricultural and environmental perspectives. Biomass Convers. Biorefin..

[65] Yaro N.S.A., Sutanto M.H., Habib N.Z., Usman A., Kaura J.M., Murana A.A., Birniwa A.H., Jagaba A.H. (2023). A comprehensive review of biochar utilization for low-carbon flexible asphalt pavements. Sustainability.

